# Kale improves bowel movements in constipated women and affects some intestinal microbes and metabolites: a pilot study

**DOI:** 10.3389/fnut.2023.1247683

**Published:** 2023-11-29

**Authors:** Yuichiro Nishimoto, Felix Salim, Yohsuke Yamauchi, Yuka Mori, Shinnosuke Murakami, Asahi Suzuki, Shinji Fukuda, Takuji Yamada

**Affiliations:** ^1^Metagen Inc., Tsuruoka, Japan; ^2^Department of Life Science and Technology, Tokyo Institute of Technology, Meguro, Japan; ^3^Q’SAI Co., LTD., Fukuoka, Japan; ^4^Institute for Advanced Biosciences, Keio University, Tsuruoka, Japan; ^5^Gut Environmental Design Group, Kanagawa Institute of Industrial Science and Technology, Kawasaki, Japan; ^6^Transborder Medical Research Center, University of Tsukuba, Tsukuba, Japan; ^7^Laboratory for Regenerative Microbiology, Juntendo University Graduate School of Medicine, Bunkyo, Japan

**Keywords:** kale, gut microbiome, gut metabolome, dietary fiber, bowel movements

## Abstract

Dietary fiber improves intestinal environments, by, among others, increasing stool frequency. Kale is a good source of dietary fiber and minerals; however, the effects of kale on the intestinal environment have not yet been evaluated. This study determined how the intestinal environment, including the intestinal microbiota and its metabolome, and stool frequency are affected by the consumption of kale, in humans. A randomized controlled crossover trial, with a 4-week consumption of kale or control food, was conducted. An integrated analysis of the intestinal microbiota and metabolome was performed, and their relationship with improvements in stool frequency was analyzed. Kale intake for 4 weeks significantly increased stool frequency and altered some intestinal microbes, such as an increase in the [*Eubacterium*] *eligens* group and a decrease in the [*Ruminococcus*] *gnavus* group. Analysis of subjects with increased stool frequency revealed that this group had smaller amounts of stool before kale intake. Our findings indicate that kale modifies certain gut microbes, such as [*Eubacterium*] *eligens* and [*Ruminococcus*] *gnavus*, and improves bowel movements, particularly in those with smaller stool amounts.

## 1 Introduction

Constipation, a common gastrointestinal disorder affecting people of all ages worldwide, is associated with higher mortality risk and incidence of coronary heart disease (1). However, those with less severe symptoms do not necessarily receive treatment. Sex, lack of exercise, and low fluid and fiber intake are risk factors for constipation (2). Improving bowel movements through diet is one of the most common strategies for treating constipation. Cruciferous vegetables (genus *Brassica*) have been studied for the prevention of dietary-associated diseases. While broccoli and cabbages are the two most popular cruciferous crops, kale is widely consumed as a green juice. Kale not only contains flavonoid glycosides, such as quercetin and kaempferol, but also high levels of insoluble dietary fiber (3, 4). The latter promotes bowel movements by increasing the bulk of stools; some of the dietary fiber is metabolized by the gut microbiota to form butyric acid, which further promotes bowel movements (5).

The effects of kale on the gut microbiota, have only been reported in mice (6, 7). Therefore, in the present study, we aimed to understand the effects of kale on the intestinal environment, including intestinal microbiota, metabolome, and stool frequency, in humans. There were few prior studies on kale intake targeting the intestinal microbiota and metabolome; therefore, a pilot randomized controlled crossover trial was conducted with a 4-week consumption of kale or control food. Since women have been reported to suffer from constipation more than men (8), this study included only women. We performed an integrated analysis of the intestinal microbiota and metabolome and analyzed their relationship with improvements in stool frequency.

We found that kale significantly improved bowel movements and observed several potentially beneficial effects, such as increasing the levels of [*Eubacterium*] *eligens* group and a decreasing those of [*Ruminococcus*] *gnavus* group of gut microbiota and increasing pimelic acid content, as a gut metabolite. Further, we analyzed the characteristics of subjects with improved bowel movements and found that the improvement was greater in subjects with smaller fecal amounts prior to kale intake. Our results provide evidence of the mechanism by which kale, increases the bulk of fecal matter in humans.

## 2 Materials and methods

### 2.1 Clinical trial

**Figure 1:**
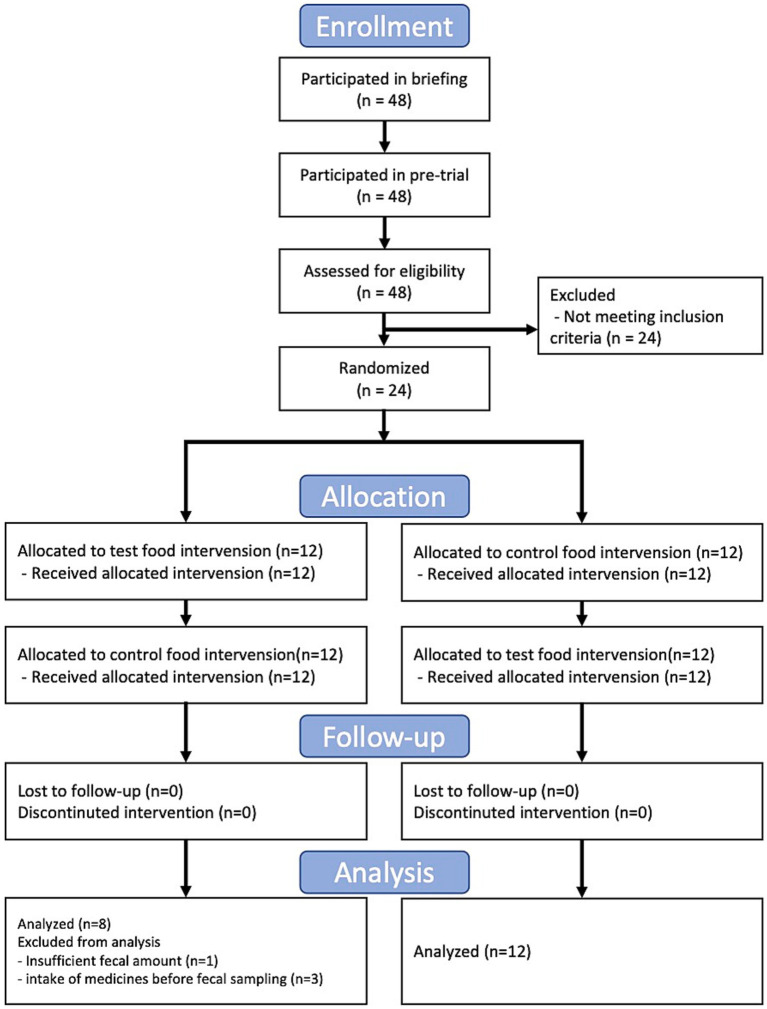
Flow diagram of the trial.

In this study, we conducted a randomized, double-blind, controlled, crossover trial for 3 months. This trial was approved by the Clinical Trial Ethics Review Committee of the Chiyoda Paramedical Care Clinic (publicly registered at UMIN-CTR, Trial number: UMIN000028734). All participants provided written informed consent. The trial included a 4-week dietary intervention period separated by a 4-week washout period (washout) (Figure 1). The test food was collard type kale, and whole kale leaf was powdered, and the control food was cornstarch and maltodextrin powder with food coloring and flavoring (Table 1). The food coloring and fragrance were processed so that the subjects could not identify the differences in taste and odor. The test or control food was mixed with 100–150 mL water and consumed twice daily. During the trial, fecal samples were collected at baseline and at 2 weeks, and 4 weeks after the dietary intervention and frozen at −20°C until gut microbiome and metabolome analysis. Female participants with constipation tendencies were recruited. The selection criteria were as follows: (1) Females between the ages of 20 to 59; (2) defecate 3 to 5 times in a week. Other detailed selection and exclusion criteria were described at Supplementary Table S1. 24 subjects were selected for the main trial and randomized according to age. All 24 subjects completed the trial; however, four subjects were excluded from further analysis for the following reasons: incomplete fecal sampling (subject 21) and intake of medication for 7 days before fecal sampling (subjects 4, 9, and 22).

**Table 1:** Composition of control food and kale.

	Control (14 g/day)	Kale (14 g/day)
Calories (kcal)	52.780	41.860
Protein (g)	0.028	2.324
Total fat (g)	0.042	0.658
Total carbohydrate (g)	13.076	9.240
Dietary fiber (g)	0.070	5.138
Sodium (mg)	6.090	39.620

### 2.2 Gut microbiome and metabolome analysis

DNA was extracted from fecal samples as previously described (9). After DNA extraction, the V1-V2 variable region of the 16S rRNA gene was amplified with the bacterial universal primers 27F-mod (5′-AGRGTTTGATYMTGGCTCAG-3′) and 338R (5′-TGCTGCC TCCCGTAGGAGT-3′) using Tks Gflex DNA polymerase (Takara Bio Inc., Shiga, Japan) (10). Amplicon DNA was sequenced using MiSeq (Illumina, United States) according to the manufacturer’s protocol. Metabolite extraction from fecal samples was performed as previously described (11). The extracted metabolites were analyzed using coupled capillary electrophoresis-electrospray ionization-time-of-flight mass spectrometry (CE-TOFMS). After peak identification, the relative area ratio to internal standards was calculated. For 79 metabolites, the amounts in stool were calculated by comparison with the reference material (Supplementary Tables S2, S3). We used QIIME2 for 16S rRNA gene analysis (version 2019.10). In the analysis pipeline, the primer base was discarded with cutadapt (option: -p-discard-untrimmed) (12). The sequence data were processed using the DADA2 pipeline for denoising and quality filtering (option: -p-trunc-len-f 230 -p-trunc-len-r 130) (13). The filtered output sequences were assigned to taxa for Silva SSU Ref NR 99 (version 132) using the “qiime feature-classifier classify-sklearn” command with default parameters (Supplementary Table S4) (14).

### 2.3 Statistical analysis

We used in-house Python programs (version 3.7.3) for statistical analysis. For beta-diversity analysis, microbiota and quantitative metabolome Bray-Curtis distances were calculated (SciPy version 1.5.1). Principal Coordinate Analysis (PCoA) was performed to visualize the resulting distance matrices (Scikit-learn version 0.21.2). In addition, the inter-time point distance was compared using permutational multivariate analysis of variance (PERMANOVA) (scikit-bio version 0.5.5). For the differential abundance analysis, each microbe and metabolite were compared using the Wilcoxon signed-rank test with Benjamini-Hochberg false discovery rate correction (SciPy version 1.5.1, and statsmodels version 0.10.1, respectively). In the comparison, differences from the baseline values were compared. In addition, microbes with a mean relative abundance below 0.001 and metabolites not detected in more than 75% of the samples were excluded from the comparison. In the correlation analysis, the Pearson correlation coefficient, Spearman rank correlation coefficient, and the test for no correlation were used (SciPy version 1.5.1). In this research, the kale effect size for each subject was defined as the response score. The response score was calculated using the following equations:

$\begin{array}{l} 2-\mathrm{week}\;\mathrm{response}\;\mathrm{score}=\left(Kale_{2\mathrm{weeks}}-Kale_{0\mathrm{week}}\right)\\ \;-\left(\mathrm{Contro}l_{2\mathrm{weeks}}-\mathrm{Contro}l_{0\mathrm{week}}\right) \end{array}$

$\begin{array}{l} 4-\mathrm{week}\;\mathrm{response}\;\mathrm{score}=\left(Kale_{4\mathrm{weeks}}-Kale_{0\mathrm{week}}\right)\\ \;-\left(\mathrm{Contro}l_{4\mathrm{weeks}}-\mathrm{Contro}l_{0\mathrm{week}}\right) \end{array}$

## 3 Results

### 3.1 Clinical trials and effects of kale on defecation events

We conducted a randomized, double-blind, placebo-controlled, crossover trial with 24 Japanese participants (Figure 1). The primary outcomes were gut microbiota and gut metabolome. The key secondary outcomes were frequency, amount, and consistency of stool defecation. The baseline clinical characteristics were similar in both groups (Supplementary Table S5). First, we compared the stool defecation data. The frequency of stool defecation significantly increased compared to that in the placebo intake period (Table 2; *p* = 0.037; Wilcoxon signed-rank test). In addition, stool amount tended to increased with intake of kale (*p* = 0.090; Wilcoxon signed-rank test). There was no adverse event in any of the subjects.

**Table 2:** Statistical test results for defecation data.

	Stool amount^*a^			Stool consistency^*b^			Defecation frequency^*c^		
	Mean	S.D.	*p*-value^*d^	Mean	S.D.	*p*-value^*d^	Mean	S.D.	*p*-value^*d^
Control 0week	13.650	4.255	0.090	3.173	0.693	0.936	0.557	0.173	0.037
Control 2weeks	16.100	5.263		3.436	0.745		0.650	0.227	
Control 4weeks	16.800	6.740		3.612	0.858		0.639	0.285	
Kale 0week	13.292	4.220		3.309	0.900		0.550	0.125	
Kale 2weeks	16.225	6.427		3.423	0.819		0.654	0.254	
Kale 4weeks	19.300	9.099		3.574	0.687		0.700	0.267	

^*a^ Stool amount was determined by counting the number of eggs. ^*b^ 1. very hard 2. hard 3. somewhat hard 4. normal 5. somewhat soft 6. soft (muddy), and 7. very soft (watery). ^*c^ Bowel movement count per day. ^*d^ *p*-value comparing control_4weeks and kale_4weeks using Wilcoxon signed-rank test. S.D., standard deviation.

### 3.2 Effects of kale on gut microbiome and metabolome composition

**Figure 2:**
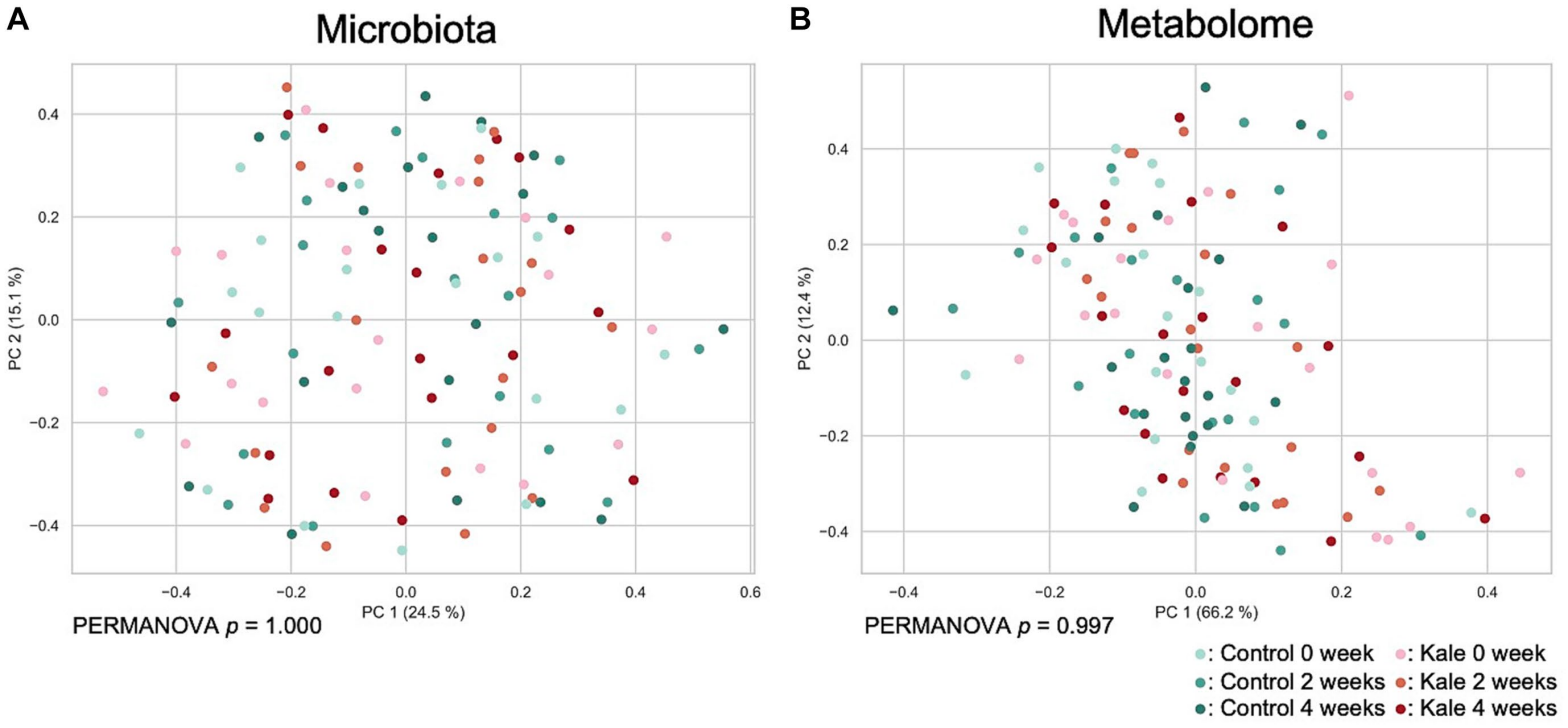
Effect of kale consumption on gut microbiota and metabolome profiles. The Bray–Curtis dissimilarity for **(A)** gut microbiota and **(B)** gut metabolome profiles among all samples was calculated and visualized using PCoA. The *p* values of PERMANOVA results for each time point are shown at the bottom of each figure.

To evaluate the effect of kale supplement intake on the gut microbiome and metabolome profiles, we performed 16S rRNA gene-based microbiota analysis and CE-TOFMS-based metabolome analysis. A total of 215 genera of gut microbes and 352 metabolites were obtained from 120 samples (Supplementary Tables S2–S4). Beta diversity analyses were performed using Bray–Curtis dissimilarity for microbiota and metabolome profiles (Figure 2). No remarkable difference due to the consumption of the kale was observed. Although PERMANOVA was performed, there was no significant difference in the gut microbiota or metabolome profiles between the timepoints (*p* = 1.000 and 0.997 for microbiota and metabolome Bray–Curtis dissimilarity, respectively).

### 3.3 Effect of kale supplement intake on gut microbes and metabolites

**Figure 3:**
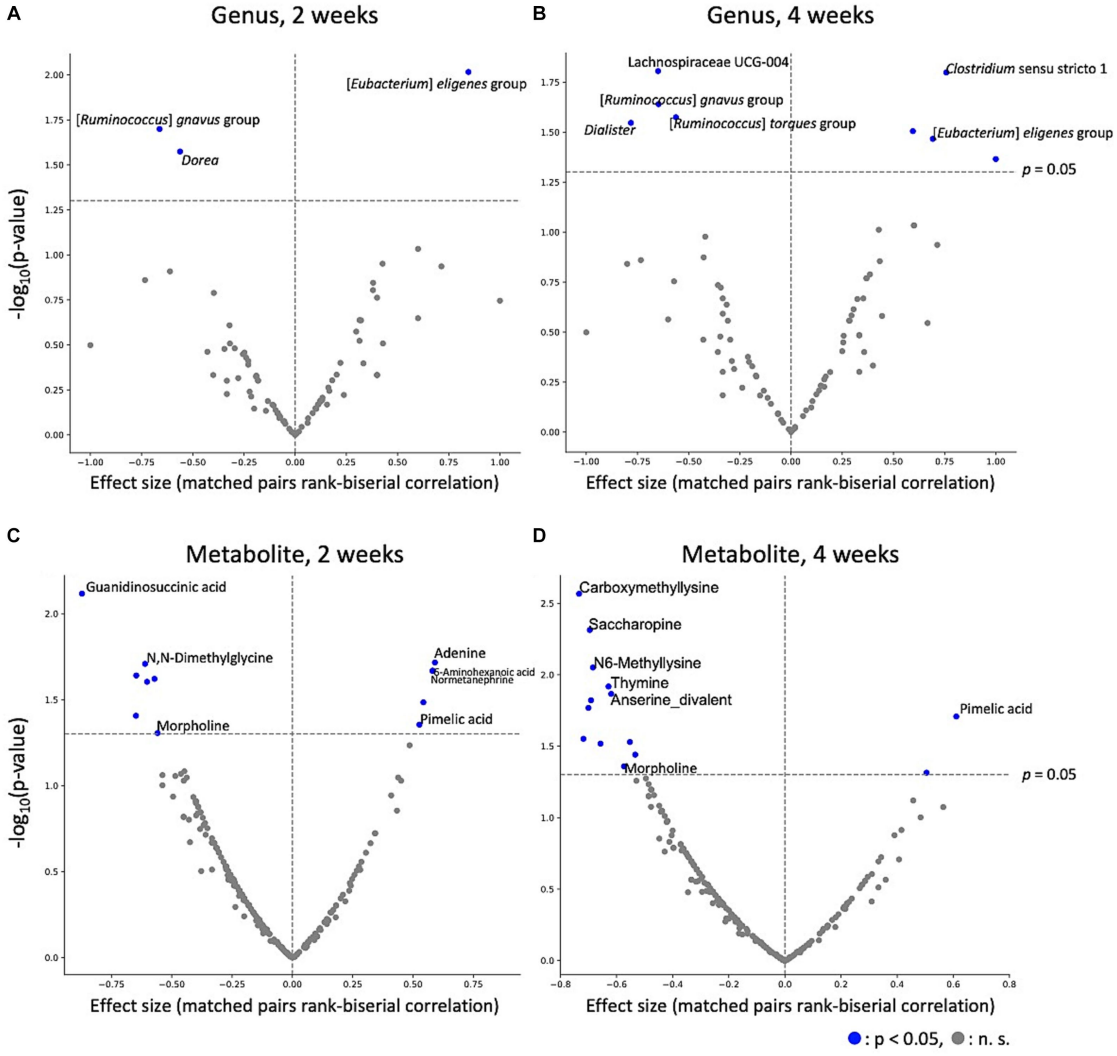
Effect of kale intake on gut microbes and metabolites. The effects of kale on **(A)** microbes at 2 weeks, **(B)** microbes at 4 weeks, **(C)** metabolites at 2 weeks, and **(D)** metabolites at 4 weeks are shown as volcano plots. The *x*-axis represents the effect size (paired rank-biserial correlation) of kale intake compared to that of the control group. The *y*-axis represents logarithm of the Wilcoxon signed-rank test *p*-value compared to that of the control group. Names of the items for which significant differences were detected at both time points, and the top five significantly different items are labeled.

To estimate the effect of kale intake on each gut microbe and metabolite, gut microbes and metabolites between after control and kale intake were compared. The results indicated that kale intake affected gut microbe abundance; [*Eubacterium*] *eligens* group was consistently higher in the kale-treated group, at both 2 and 4 weeks, while that of [*Ruminococcus*] *gnavus* group was consistently lower (Figures 3A,B). Analysis of gut metabolite abundance showed that pimelic acid content was consistently higher while morpholine content was consistently lower at both 2 and 4 weeks of kale intake (Figures 3C,D). After false discovery rate (FDR) correction, significant differences were detected in the absence of microbes and metabolites (Supplementary Tables S6, S7). These results indicate that kale affects some gut microbes and metabolites.

### 3.4 Effect of kale supplement intake on gut microbes and metabolites

Previous studies have reported that individual responses to meals or drugs are partly attributed to differences in gut microbiota. Here, we defined response scores from stool frequency improvement upon kale supplementation and explored which gut environmental factors correlated with the response.

**Figure 4:**
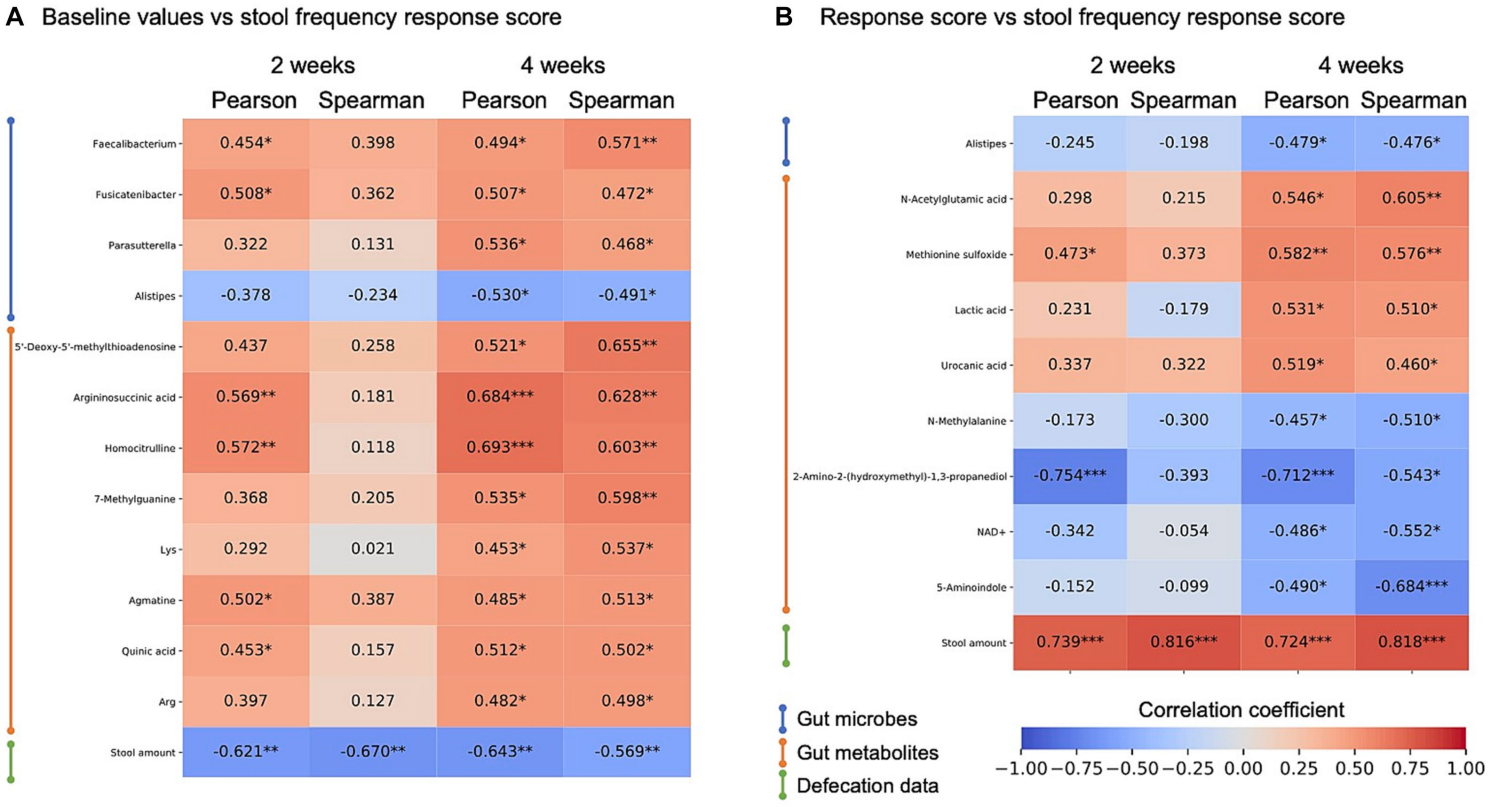
Heatmap of correlation analysis of stool frequency response score. Correlation analysis between stool frequency response scores and gut enviromental features of **(A)** baseline value correlations and **(B)** response score correlation, and its correlation coefficient is shown by heatmap. Items showing significant correlations were extracted at 4 weeks of intake. Heatmap rows were sorted by data type and 4-week Spearman correlation coefficient value. *, *p* < 0.05; **, *p* < 0.01; ***, *p* < 0.001, *p*-values were calculated by correlation coefficient ‘s no correlation test.

First, the effect of baseline features was analyzed. Correlation analysis between the stool frequency response score and microbes, metabolite abundance, and stool defecation data at baseline showed a consistent negative correlation between the response and baseline stool amount (Figure 4A; Supplementary Table S8). This indicates that kale improves defecation frequency, especially in those with low stool amounts. Many other bacteria and metabolites were significantly correlated, but there was no consistent correlation between weeks 2 and 4. Next, we defined the response scores for all microbes, metabolites, and defecation data and calculated the correlation between these response scores and stool frequency response score. In this analysis, a consistent positive correlation was detected between stool frequency and stool amount response score. For gut metabolites, an increase in lactic acid correlated with an increase in the frequency of defecation, albeit only for 4 weeks (Figure 4B).

## 4 Discussion

In this study, the effects of kale intake on defecation frequency, gut microbiota and metabolome were analyzed and potential links between the gut microbiota and metabolome and the effect of kale juice intake on defecation frequency were identified.

Using PERMANOVA and PCoA of the gut microbiota and metabolome, we observed minimal difference in the overall gut microbiota or metabolome composition following kale intake (Figure 2). However, Wilcoxon signed-rank tests showed an increase in the [*Eubacterium*] *eligens* group and a decrease in the [*Ruminococcus*] *gnavus* group abundance following kale intake. *Eubacterium eligens* has been reported to utilize dietary fiber, especially pectin, as an energy source (15). In addition, it has been reported to strongly promote the production of the anti-inflammatory cytokine interleukin-10 in *in-vitro* cell-based assays (15). *Ruminococcus gnavus* is a mucolytic bacteria, with reports of its relations with inflammatory bowel disease (16, 17). However, *R. gnavus* has been reported to possibly have beneficial and harmful effects on the host, depending on the subspecies (18). Therefore, determination of whether its decrease was beneficial to the host was difficult. These results also suggest that kale consumption may reduce intestinal inflammation through increased [*Eubacterium*] *eligens* group and decreased [*Ruminococcus*] *gnavus* group. The fecal metabolite results showed that pimelic acid was consistently higher upon kale intake than control food. Pimelic acid is a precursor of biotin (vitamin B_7_), a gut microbiota-derived compound that is essential for energy metabolism in humans (19). Only some bacteria can produce biotin, whereas those that cannot produce biotin express free biotin transporters. Biotin is necessary for the growth and survival of bacteria. Given that some bacteria compete for biotin, the control of biotin levels in the gut may affect the proportion of some bacteria. The association between morpholine and the intestinal environment has not been previously reported.

A significant improvement in stool frequency was observed after 4 weeks of kale intake compared with control food. In a previous study, insoluble fiber was shown to have a high water-holding capacity that increased the fecal bulk, thereby increasing the frequency of defecation (20). Kale contains a large amount of insoluble fiber (4), which may be responsible for this effect. In addition, improvement in stool frequency was most profound in subjects with low stool amounts at baseline. This possibly indicates that the water retention effect of kale is effective in subjects with low fecal volume (low fecal water content). The findings suggest that kale intake could be beneficial for alleviating mild constipation by increasing stool bulk with dietary fiber and leading to an increase in stool frequency.

Correlation analysis showed that several gut microbes and fecal metabolites correlate with the subjects’ responses to kale intake. However, we did not find any association consistent with the baseline value/response score for the fecal amount. Other gut microbiota and metabolites may be markers of fecal amounts. However, there was a positive correlation between increased lactic acid levels and increased defecation frequency after 4 weeks of kale intake (Figure 4B). Lactic acid is an intermediate in propionic acid production (21). Propionic acid and other short-chain fatty acids are associated with peristalsis through the production of serotonin and calcitonin gene-related peptides (CGRP) (22). Therefore, increased lactic acid levels could lead to increased stool frequency.

The study has some limitations. Gut microbes and their metabolites are complex parameters consisting of several different bacterial microbes and metabolites. FDR correction is necessary when comparing items; however, after FDR correction, no significant differences were found in any of the microbes and metabolites. This is because many items were analyzed simultaneously. In addition, the small sample size also contributed to the difficulty in drawing conclusions from this study alone. This study was a pilot study, hence further validations in another cohort or mouse model may be necessary, especially considering the effects of kale on gut microbes and metabolites.

As the conclusion, this randomized controlled study provided novel insights into the effects of kale on the intestinal environment and defecation events in a Japanese population. Kale intake significantly increased stool frequency, [*Eubacterium*] *eligens* group abundance, and pimelic acid amount, and decreased [*Ruminococcus*] *gnavus* group abundance and morpholine amount. In addition, the improvement in stool frequency was dependent on the baseline fecal amount of the subjects. Our findings revealed the impact of kale on the intestinal environment and shed light on stratified healthcare considering the intestinal environment.

## Data availability statement

The datasets presented in this study can be found in online repositories. The names of the repository/repositories and accession number(s) can be found at: https://www.ddbj.nig.ac.jp/, DRA016616.

## Ethics statement

The studies involving humans were approved by Chiyoda Paramedical Care Clinic. The studies were conducted in accordance with the local legislation and institutional requirements. The participants provided their written informed consent to participate in this study.

## Author contributions

AS and SF: conceptualization. YN: data curation, formal analysis, and visualization. YM: investigation. YN and FS: writing – original draft. YN, FS, YY, YM, SM, AS, SF, and TY: writing – review & editing. All authors contributed to the article and approved the submitted version.
